# Implementation of Refugees' Inclusion in National Viral Hepatitis B and Hepatitis C Screening Campaign in Mahama Refugee Camp, Rwanda

**DOI:** 10.9745/GHSP-D-21-00349

**Published:** 2022-04-28

**Authors:** Françoise Nyirahabihirwe, Innocent Kamali, Dale A. Barnhart, Jean de la Paix Gakuru, Tumusime Musafiri, Dina Denis Rwamuhinda, Placide Mutabazi, Stephanie Mukayirabuka, Jean Damascene Makuza, Noor Kassim, Joel M. Mubiligi, Jean d'Amour Ndahimana, Fredrick Kateera

**Affiliations:** a Partners In Health- Rwanda/Inshuti Mu Buzima, Rwinkwavu, Rwanda.; b Department of Global Health and Social Medicine, Harvard Medical School, Boston, MA, USA.; c Save the Children International, Kigali, Rwanda.; d Alight, Kigali Rwanda.; e Rwanda Biomedical Centre, Kigali, Rwanda.; f School of Population and Public Health, University of British Columbia, Vancouver, Canada.; g United Nations High Commissioner for Refugees, Kigali Rwanda.

## Abstract

**Introduction:**

The World Health Organization has called for the elimination of hepatitis B virus (HBV) and hepatitis C virus (HCV) as public health threats by 2030. In response to the United Nations High Commissioner for Refugees requests, Rwanda became the first country to include refugees in its national viral hepatitis prevention and management program in 2019. We used secondary data to describe the implementation of the first HBV and HCV screening program among refugees in Rwanda.

**Methods:**

Rapid diagnostic tests were used to screen for HBV surface antigen (HBsAg) and HCV antibody (anti-HCV). We used routine data collected during the HBV and HCV mass screening campaign among Burundian refugees living in Mahama camp and program records to estimate the screening coverage, the prevalence of HBV and HCV, and the cost of the campaign.

**Results:**

Over 28 days in February and March 2020, 26,498 unique individuals were screened for HBV and HCV, reflecting a screening coverage of 77.9% (95% confidence interval [CI]=76.5%, 78.4%). Coverage was greater than 90% among women aged 30–64 years, but younger age groups and men were less likely to be screened. On average, 946 clients were screened per day. The prevalence of anti-HCV was 1.1% (95% CI=1.0%, 1.3%), and the prevalence of HBsAg was 3.8% (95% CI=3.6%, 4.0%). We estimate that the total cost of the campaign was US$177,336.60, reflecting a per-person-screened cost of US$6.69.

**Conclusion:**

Conducting a mass screening was a feasible and effective strategy to achieve high screening coverage and identify refugees who were eligible for HBV and HCV treatment. This screening program in the Mahama refugee camp can serve as a reference for other refugee camps in Rwanda and elsewhere.

## Abstract (precis)

Conducting a high-quality mass screening campaign for Hepatitis B virus and Hepatitis C virus was a feasible, effective, and low-cost strategy to integrate refugees into Rwanda's national hepatitis prevention and management program.

## Key Messages

- Conducting a mass screening campaign for Hepatitis B and Hepatitis C viruses was a feasible, effective, and low-cost strategy to integrate refugees into Rwanda's national hepatitis prevention and management program.
- Coordination across national-, district-, and community-level stakeholders, as well as real-time data analysis, are critical for implementing a well-coordinated, high-quality campaign and supported high screening coverage.
- This campaign provided an opportunity to identify clients who were eligible for treatment and to provide educational counseling on prevention measures among people who screened negative. Policy makers should explicitly include refugees and other displaced persons in their national hepatitis elimination and control strategies.

Résumé en français à la fin de l'article.

## INTRODUCTION

Viral hepatitis affects 325 million people, and hepatitis B (HBV) and hepatitis C (HCV) are the leading causes of hepatitis-related deaths worldwide.^1^ Although viral hepatitis causes more than 1 million deaths every year, with Asia and Africa being the most affected regions, most people living with HBV or HCV do not know their status.^2^ These regions are also home to a large and growing number of migrants and refugees. Nearly 26 million refugees live in a foreign country and Asian and African countries host 84% of all refugees.^3^ Although refugees in the United States, Turkey, and Iraq have been reported to face an elevated risk of viral hepatitis,^4^^–^^6^ efforts to identify and treat migrants and refugees with HBV and HCV are often limited to small outreach programs and focused in urban areas.^7^ Lessons learned from previous HIV programs suggest that integrating refugees into national HBV and HCV screening and treatment programs could both promote health equity and support the host countries' overall hepatitis responses.^8^

Among Rwandans aged older than 25 years, the prevalence of HBV surface antigen (HBsAg) is 3.9% and of HCV antibody (anti-HCV) is 6.8%.^9^^,^^10^ In 2018, the Rwandan Government launched the national HCV elimination plan, which offers free hepatitis screening services for Rwandan residents aged 15 years or older through mass screening campaigns and provides free treatment to all eligible cases identified with HBV and HCV.^11^ Rwanda is also home of more than 145,000 refugees, including 64,000 Burundian refugees living in the Mahama refugee camp.^12^ In November 2019, in response to a request from United Nations High Commissioner for Refugees (UNHCR), the Rwandan Ministry of Health through Rwanda Biomedical Centre (RBC) approved the inclusion of refugees in the national hepatitis program.^12^ Refugees had not been previously included in the national hepatitis program, and a program to implement this policy did not exist. It was not known whether it would be feasible to implement mass screening campaigns, which had previously been implemented for the general population, in a refugee camp setting.

Refugees had not been previously included in the national hepatitis program, and a program to implement this policy did not exist.

We describe the implementation of a mass hepatitis screening campaign conducted in the Mahama refugee camp, including the flow of clients through the screening and confirmatory testing processes, screening coverage, and the cost of the screening program. By sharing our programmatic experiences, we hope to provide information to policy makers and stakeholders seeking to include refugees and other displaced persons in their national hepatitis elimination and control strategies.

## HEPATITIS SCREENING PROGRAM

### Standard of Care for Hepatitis in Rwanda

Per Rwandan national guidelines, individuals aged 15 years and older are eligible for HBV and HCV screening. Voluntary screening is commonly provided through mass screening campaigns, where clients are tested for HBsAg and anti-HCV using rapid diagnostic tests (RDTs). Clients with positive screening results provide a venous blood sample for polymerase chain reaction-based viral load (VL) testing.^13^

Per national guidelines, clients with a detectable VL for HBV receive further clinical evaluations to assess for eligibility to initiate antiretroviral treatment, which is typically a lifelong course of tenofovir 300 mg. Clients with a detectable VL for HCV receive a course of directly acting antivirals consisting of daclastasvir 60 mg, sofosbivir 400 mg for 12 weeks (referred to as 3 months) for clients without evidence of decompensated cirrhosis or for 24 weeks (referred to as 6 months) among those with aspartate aminotransferase to platelet ratio index score (APRI) of 2 or higher, which has been shown to result in a sustained virological response, or cure, for more than 90% of cases.^14^

### Setting and Stakeholders

The Mahama refugee camp, located in Kirehe district of Rwanda, was established in 2015 to host Burundian refugees. It is the sixth largest and most recent refugee camp with 64,000 residents. It is managed by the Ministry of Emergency Management (MINEMA), which works closely with UNHCR and the refugees' executive committee representatives, a group of Mahama residents and refugees who are elected to represent the camp residents' interests. Save the Children and Alight are key UNHCR partners who support health in the camp by managing 2 primary-level health centers. Community health workers (CHWs) support the clinics and other health-related activities in the community. Health care for residents is also supported by the Ministry of Health and Partners In Health/Inshuti Mu Buzima (PIH/IMB), a nongovernmental organization that has supported access to health care and health system strengthening in Rwanda since 2005. In collaboration with UNHCR and the health partners from the camp, PIH/IMB organized and conducted the HBV and HCV mass screening campaign. According to data provided by MINEMA, approximately 34,000 (57%) of Mahama residents were aged 15 years and older and eligible to be screened for HBV and HCV.

### Leadership Engagement and Community Mobilization

The screening campaign was preceded by a series of preparatory meetings with stakeholders to understand the setting, define roles and responsibilities, and develop coordination mechanisms for implementation and follow-up. To facilitate crowd management, the leadership team developed a schedule designating specific days and screening locations for each of the 18 villages within the camp. This schedule was shared with the CHWs who used different strategies, including door-to-door visits, to prepare households for the screening and to mobilize individuals to attend screening on their villages' scheduled day. Statements from the refugee executive committee, communiqués in churches, posters, and announcements via megaphones were also used to sensitize the population to attend the screening.

### Staffing and Training

A 40-person team composed of 12 data clerks, 14 nurses, and 14 laboratory technicians received an intensive 2-day training. Data clerks were trained to use paper-based laboratory request forms and the Research Electronic Data Capture (REDCap) mobile application, a tablet-based data collection device, to collect participants' demographic data and contact information. Laboratory technicians were trained on Rwanda's hepatitis C and B screening algorithm; use of RDTs; interpretation of RDT results; and venous blood sample collection, storage, and transport.

Nurses were trained on national guidelines for viral hepatitis prevention and management in Rwanda, educating clients on hepatitis, performing capillary and venous blood collection, and conducting pre- and post-test counseling. All cadre of workers learned about ethics and behavior in the camp. The training of the screening team was conducted primarily in English with supplementary translations in Kinyarwanda, Rwanda's local language, to ensure full understanding. CHWs from the camp also received a half-day orientation on their roles as community mobilizers and support staff at screening sites. The orientation of CHWs was conducted in Kinyarwanda, which is mutually intelligible by speakers of Kirundi, the local language of Burundi.

### Screening Activities

On February 5, 2020, the campaign was publicly launched and the event was open to all Mahama refugee camp residents. The event included musical and dancing entertainment, remarks from leaders of stakeholder organizations, and testimony from a Mahama camp resident who had been diagnosed with HCV in 2000 and had worked with UNHCR to advocate for hepatitis care and treatment in the camp. These remarks emphasized the importance of including refugees in the national HBV and HCV prevention and management program and the long-term complications of hepatitis.

At the campaign launch, a Mahama camp resident advocated for hepatitis care and treatment in the camp, emphasizing the importance of including refugees in hepatitis programs.

At the launch event, 127 selected individuals, including the refugee executive committee members, people living with disabilities, CHWs, and peer educators, were screened. Screening was conducted in 3 different community locations located in the camp—a library, a community center, and a discontinued quarantine facility. Each site had multiple screening “teams” each consisting of at least 1 data entry clerk, 1 lab technician, and 1 nurse. Lab technicians and counselors provided participants with group education on the importance and benefits of screening and modes of HBV and HCV transmission and prevention. Participants were assured of privacy and confidentiality and participation in the screening was voluntary. Before testing, participants visited a registration desk where contact information, sociodemographic information, and risk factors for HBV and HCV infections were recorded. The registration form used for data collection was a digital adaption of the national form used during previous screening campaigns of the Rwandan general population. Data were collected using tablets loaded with REDCap mobile app software. During registration, each participant was also provided with a laboratory request form, which was used to record RDT results. Then, capillary blood samples were collected and tested on RDT. We used RDTs manufactured by Standards Diagnostics Ltd, Kyonggi-do, Korea, to detect HBsAg with a sensitivity of >99% and specificity of >99% and anti-HCV with a sensitivity of 96.7% and specificity of 98.9%.^15^

The participants had to wait 20 minutes for RDT results. Participants were given their test results individually and received counseling on HBV and HCV prevention. Results from the RDTs were transferred from paper laboratory request forms to tablets, and data on the tablets were uploaded to a secure server and assessed for quality. These data were used to monitor screening coverage. Finally, a summary report was shared with stakeholders listing the number of people screened and tested positive. Among participants who tested positive, a 4–5 ml venous blood sample for HBV deoxyribonucleic acid (DNA) and/or HCV ribonucleic acid (RNA) was collected in ethylene diamine tetra-acetic acid (EDTA) tubes for VL testing. Collected blood samples for VL testing were transported the same day to Rwamagana provincial hospital, the nearest VL testing hub. To facilitate prompt analysis of the samples, hospital lab technicians were provided with small 1-time transfer incentives (US$217.39) to compensate them for overtime or weekend work. COBAS AmpliPrep/COBAS TaqMan HCV and HBV Test, V.3.0: Quantitative (Roche) with a lower limit of quantification of 15 IU/mL was used for HCV RNA and HBV DNA test, respectively.

### Developing Stockpiles

At the start of screening, all medical commodities and supplies were stored in PIH/IMB's main warehouse near the Kirehe district hospital. Because this strategy sometimes resulted in stock-outs of key supplies, we worked with collaborators from Save the Children and Alight to establish a small stockpile of supplies at each of the 3 screening sites within the camp. These stockpiles were resupplied by the main store. We designated a team leader at each screening site who coordinated the screening process and requested additional materials and commodities on a weekly basis.

### Screening in Schools

During the screening program, we assessed screening coverage by comparing the number of clients screened to the number of recorded Mahama residents aged older than 15 years. Coverage was assessed both overall and by age and sex. A midway analysis of registration data revealed that screening coverage was low among clients aged 15–25 years. Because school-aged youth attend nearby schools located outside the camp, they were absent from the camp during our weekday screening campaign. To overcome this challenge, we consulted with the heads of 2 nearby schools and were advised to conduct screening at schools during school days. Four screening teams were mobilized to conduct screening at 2 schools, which predominately serve refugees. All students older than age 15 years, including Rwandans, were able to be screened, but Rwandan students' results were recorded in a separate database. The flow of the screening activities at both schools was otherwise the same as the screening in the camp.

### Linkage to Care

As part of the cascade of viral hepatitis care, VL results were returned to the 2 primary-level health facilities in the Mahama refugee camp. The infectious disease nurse at each facility collaborated with CHWs to contact and schedule appointments with the clients. Once at the health facility, clients were treated in accordance with Rwandan national guidelines for the management of HBV and HCV. Clients with HBV were eligible for treatment if their VL was ≥20,000 IU/ml, if they had a detectable VL and APRI≥2, had HIV, or if they have been clinically diagnosed with cirrhosis. Treatment for HBV is lifelong and consists of tenofovir or entecavir. HBV clients who were not yet eligible for treatment received follow-up VL tests every 6 months to assess treatment eligibility. All clients with detectable VLs were eligible for HCV treatment, which consists of directly acting antivirals for 12 weeks among clients without evidence of decompensated cirrhosis or for 24 weeks among those with APRI≥2. Twelve weeks after the completion of treatment, clients were reassessed for sustained virologic response. Those with undetectable VLs were considered cured while others were eligible for second-line treatment.

At the time of treatment initiation, 2 clinicians at the Mahama camp health facilities had previously been certified to provide hepatitis treatment, but these clinicians were inexperienced at hepatitis treatment. Consequently, PIH/IMB sent 6 staff (2 clinicians certified in prescribing hepatitis drugs, 2 additional nurses, and 2 lab technicians) to support the camp's health facilities during client initiation. These extra staff members helped to accommodate the increased workload associated with initiating many new clients on treatment and provided mentorship to the Mahama health facility staff to ensure that they could independently provide hepatitis care in the future. After the initial treatment initiation, 1 nurse stayed at each clinic to continue to support linkage and follow up for an additional 6 months. We plan to report fully on treatment outcomes in future studies.

After viral load results were returned, clients who were eligible for treatment were treated in accordance with Rwandan national guidelines.

## METHODS

Data from the REDCap screening database were used to assess screening coverage and outcomes. Duplicates were removed before analysis. We calculated the screening coverage, defined as the number of clients screened divided by the number of eligible Mahama residents, and exact 95% confidence intervals (95% CIs) for the overall population of all residents aged older than 15 years and point estimates for screening coverage disaggregated by age and sex. For screening coverage calculations, the denominator of number of eligible Mahama residents was determined using programmatic records from Mahama camp, which includes data on age and sex. We calculated the prevalence of HBsAg and anti-HCV, presented with the corresponding exact 95% CIs for the overall population. We also estimated the VL positivity rates for both HBV and HCV as the proportion of people with detectable VL results among people with VL test results returned. We conducted all statistical analyses using Stata v.15.1 (Stata Corp, College Station, TX, USA).

To estimate the cost of screening, we used an ingredients-based costing from the perspective of the screening provider. We categorized costs as belonging to either overhead and administration, screening staff, staff training, program launch, transport, VL testing, data collection tools, supplies, and materials. We estimated the costs of screening staff, training activities, launching, transport, VL testing, medical commodities, and supplies from invoices and program budgets. We estimated the cost of administration by allocating the salary of permanent members of the PIH/IMB team proportionally to the time allocated to the screening project. We calculated the sample transportation cost, which used cars from the PIH/IMB fleet, by annualizing the expense of a car assuming an annual discount rate of 10%, a useful lifespan of 15 years, and a purchase price of US$76,087. We calculated the cost of the driver by allocating the salary of a PIH driver proportionally to the time allocated to the screening project. We also calculated the daily cost of tablet use by annualizing the expense of tablets assuming an annual discount rate of 10%, a useful lifespan of 2 years, and a purchase price of US$350. All conversions of annual to daily costs assumed 260 working days per year and all conversions of RWF to US$ assumed an exchange rate of 1 US$ to 920 RWF. We report the total cost; the cost per person screened, which was calculated by dividing the total cost by the number of unique individuals screened; and the cost per case detected, which was calculated by dividing the total cost by the number of individuals who screened positive for either HBsAg or anti-HCV. We performed all costing analyses using Microsoft Excel.

### Ethics Approval

Inshuti Mu Buzima Research Committee (IMBRC), Rwinkwavu Rwanda, and Rwanda National Ethics Committee (RNEC) 015/RNEC/2020 approved the study. The Ministry of Health through its implementing agency, RBC, also approved the screening campaign. Participants gave their oral consent for screening. However, because this study used data collected as part of routine clinical practice, we did not obtain informed consent.

## KEY FINDINGS

**FIGURE 1:**
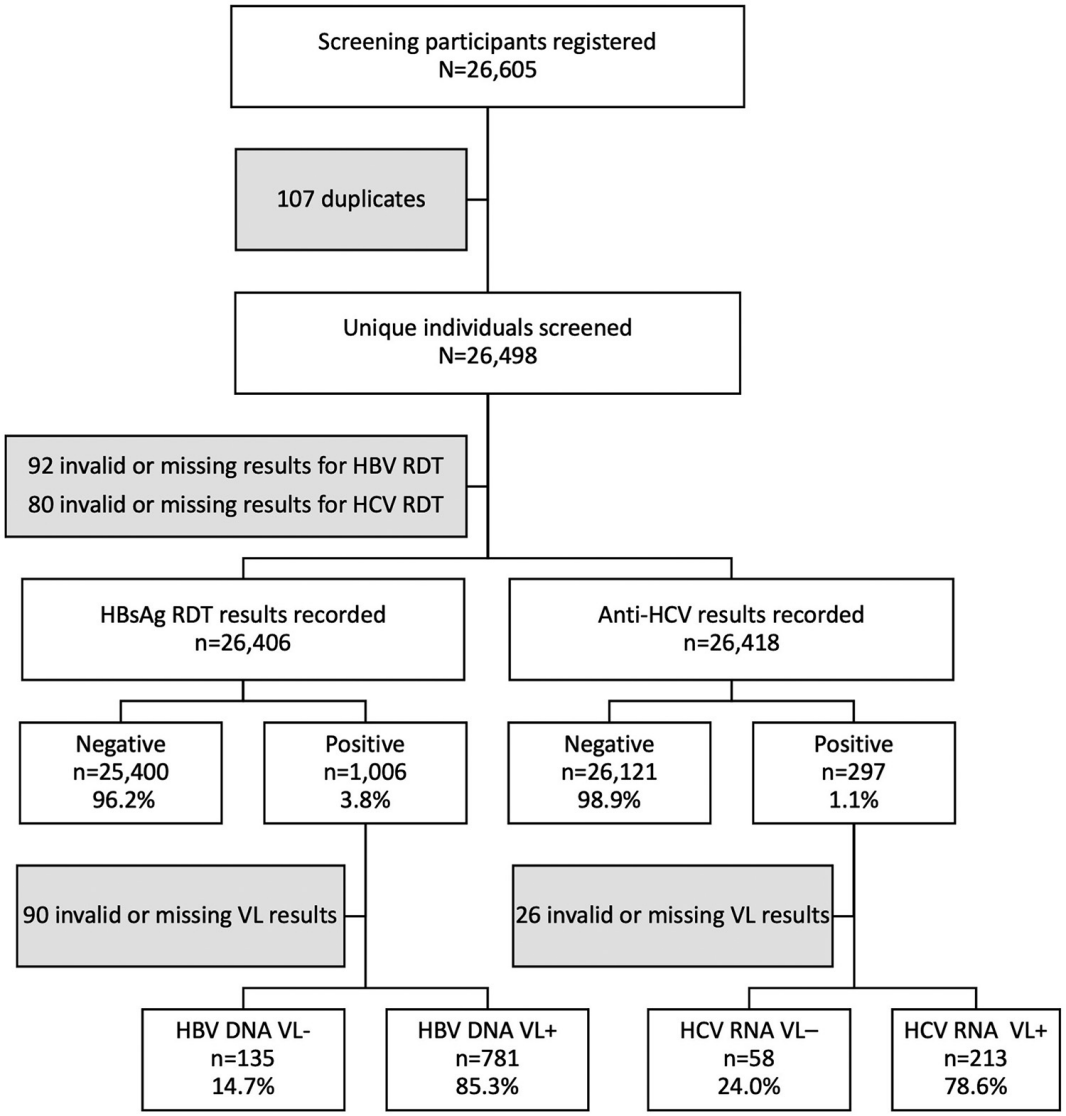
Flow Chart for Mass Screening Campaign for Hepatitis B and Hepatitis C in Mahama Refugee Camp, Rwanda Abbreviations: Anti-HCV, hepatitis C antibody; HBV, hepatitis B virus; HbsAg, hepatitis B surface antigen; HCV, hepatitis C virus; RDT, rapid diagnostic test; VL+, detectable viral load; VL-, undetectable viral load. ^a^A positive HbsAg RDT reflects a previous exposure to HBV. ^b^A detectable HBV VL of >20 copies/ml (HBV DNA VL+) reflects an ongoing infection. ^c^A positive anti-HIV RDT reflects a previous exposure to HCV. ^d^A detectable HCV VL of >15 copies/ml (HCV DNA VL+) reflects an ongoing infection.

From February 5 to March 13, 2020, we provided 26,605 HBV and HCV screening consultations, which reflected 26,498 unique individuals (Figure 1). Among them, 12,148 were men, 14,138 were women, and 212 had no sex recorded. Although staff reported cases where a single individual presented for screening on multiple occasions, in practice, only 107 duplicate individuals were identified in the database, suggesting that repeat screening was not common and usually occurred among people who had screened positive and sought a second test to confirm their test results. For both HBsAg and anti-HCV, test results were recorded for more than 99.6% of individuals.

During a 28-day campaign, we screened 26,498 individuals for HBV and HCV.

Overall, 1,006 individuals screened positive for HBsAg, reflecting a prevalence of 3.8% (95% CI=3.6%, 4.0%). As of March 5, 2021, 916 of these clients had received VL test results (91.0%) and 781 (85.3%, 95% CI=82.8%, 87.5%) had a detectable VL requiring linkage to care. For anti-HCV, 297 (1.1%, 95% CI=1.0%, 1.3%) screened positive. VL results were available for 91.2% of clients, and 78.6% (95% CI=73.2%, 83.3%) of individuals with valid test results had detectable VL and were eligible for treatment assessment. Nine individuals were coinfected with both HBV and HCV.

**FIGURE 2:**
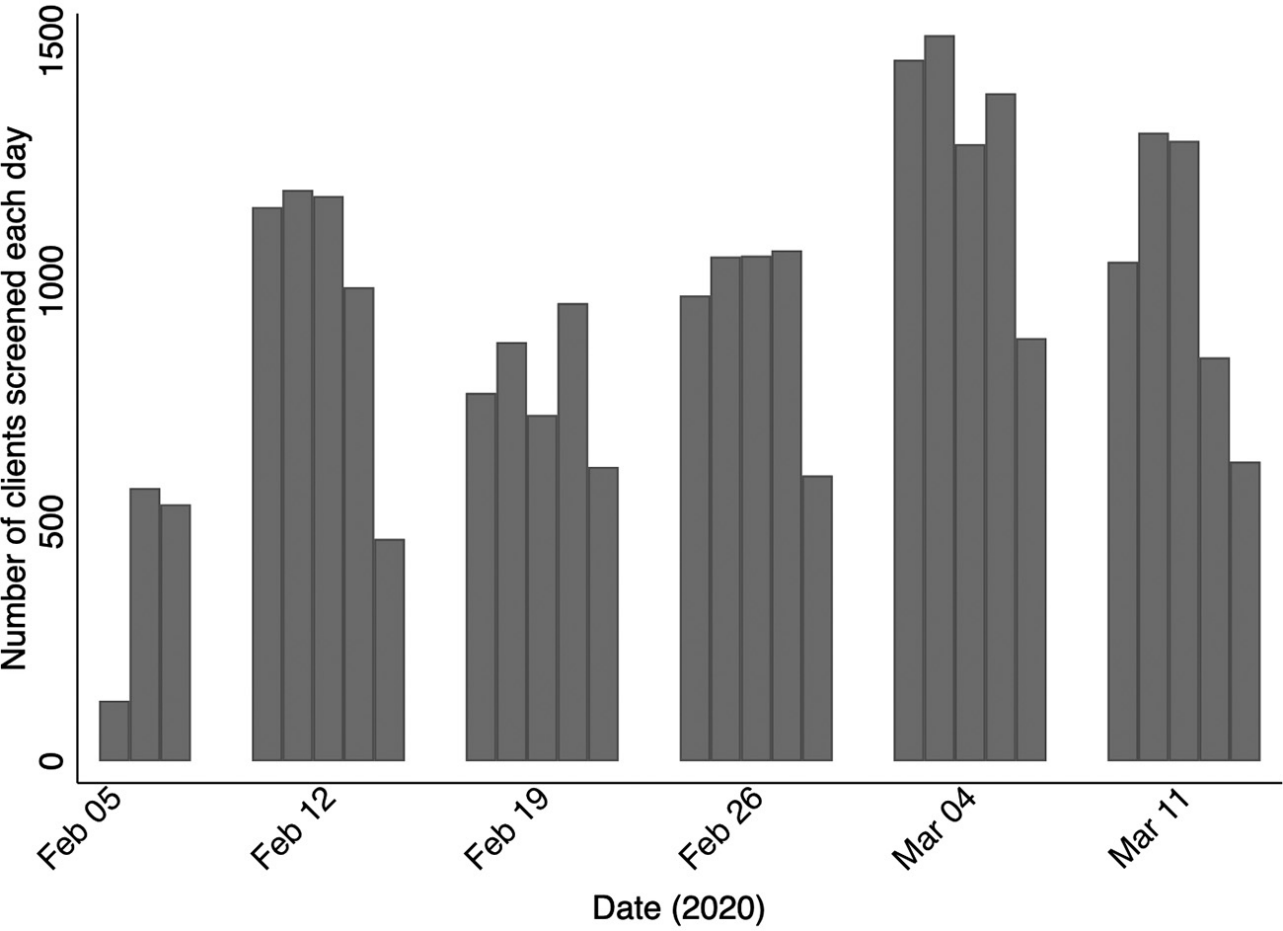
Number of Clients Screened for Hepatitis B and Hepatitis C Per Day in Mahama Refugee Camp, Rwanda^a^ ^a^Average clients screened per day was 946. Total clients screened was 26,498.

**FIGURE 3:**
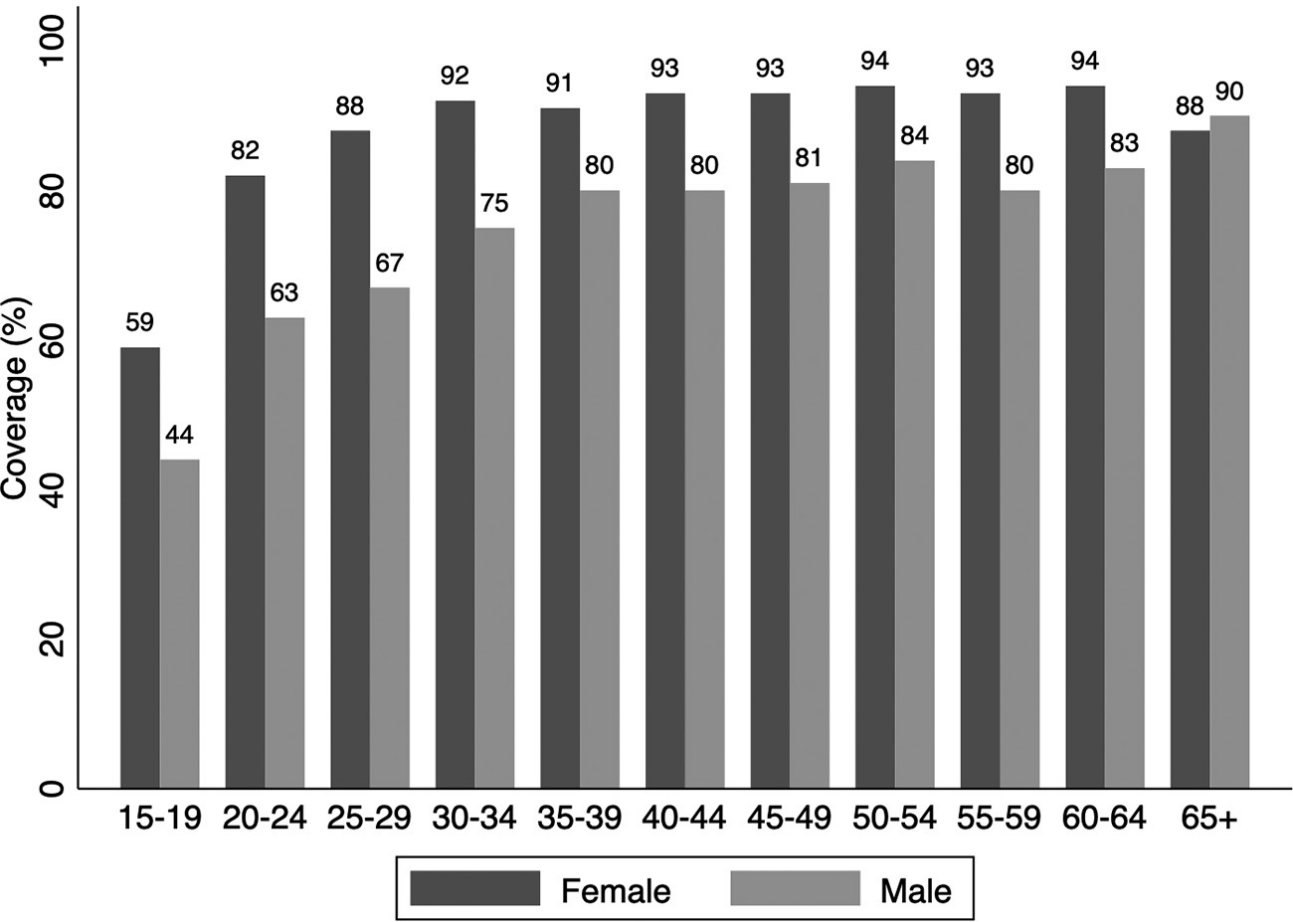
Hepatitis B and Hepatitis C Screening Coverage by Sex and Age, in Mahama Refugee Camp, Rwanda

During the screening program, an average of 946 clients were screened per day (Figure 2). Based on demographic data provided by MINEMA, this reflected an overall screening coverage among refugees aged 15 years and older of 77.9% (95% CI=76.5%, 78.4%). Coverage was greater than 90% among women aged 30–64 years. In general, younger age groups and men were less likely to be screened (Figure 3).

The overall cost of this activity was estimated to be US$177,336.60 (Table). This translated to a cost of US$6.69 per person screened or US$136.11 per case of viral hepatitis identified. Major drivers of program cost were medical supplies (US$62,916.26) and screening staff (US$55,682.26) followed by VL testing (US$21,445.65), transportation (US$13,184.15), and administrative costs (US$12,042.21).

**TABLE.** Cost of Providing Hepatitis B and C Screening to Mahama Refugee Camp, Rwanda

	Quantity	Unit Cost, US$	Total, US$
Administration and Overhead^a^			12,042.21
Screening Staff			
Clinical staff	40^b^	646.95	51,756.17
Community health worker	40^c^	3.26	3,652.17
Security guard	3^c^	3.26	273.91
		Subtotal	55,682.26
Supplies and materials			
HBV rapid diagnostic tests	27,192	1.10	29,911.20
HCV rapid diagnostic tests	27,192	1.10	29,911.20
Laboratory request form	28,494	0.03	774.29
Alcohol swabs	25,894	<0.01	225.17
Blood lancet	35,420	<0.01	308.00
Cotton rolls (500 mg)	17	1.52	25.87
Gloves	36,696	0.02	777.80
Needles	1,679	0.04	63.88
EDTA tube	1,679	0.05	91.25
Waste bags	280	0.65	182.61
Dust bins	10	41.85	418.48
Safety boxes	58	3.04	176.52
Cooler boxes	2	25.00	50.00
		Subtotal	62,916.26
Viral load testing^d^			21,445.65
Training			2,418.48
Launch materials			3,550.00
Transport^e^			13,184.15
Data collection tablets^f^			558.46
Refreshment			5,539.13
Total cost of program			177,336.60
Total cost per person screened			6.69
Total cost per case detected			136.11

Abbreviations: HBV, hepatitis B virus; HCV, hepatitis C virus; EDTA, ethylene diamine tetra-acetic acid.

a Cost of administration and overhead was estimated by allocating annual gross of salaries of infectious disease team members proportionally to the total number of days each permanent staff member dedicated to managing this project.

b 2 months' duration.

c 28 days' duration.

d In Rwanda, the unit cost of each viral load test is US$16.30 (15,000 RWF). Some additional incentives were provided to laboratory technicians at the hospital to compensate them for additional labor during the campaign.

e Annualized cost of a vehicle assumes a purchase price of US$76,086.95, an annual discount rate of 0.1, and a useful lifespan of 15 years.

f Annualized cost of tablet assumes a purchase price of US$350, an annual discount rate of 0.1, and a useful lifespan of 2 years.

## DISCUSSION

Over a 28-day campaign, we screened more than 26,498 refugees for HBV and HCV and achieved 77.9% coverage among eligible residents of the Mahama camp. The prevalence of HBsAg among screening campaign participants aged 15 years and older in Mahama (3.8%) is higher than what has been reported in Rwanda's recent population-based survey conducted among a nationally representative sample of individuals aged 15 to 64 years (2.0%)^16^ but comparable to a previous estimate from mass screening campaigns among the Rwandan general population aged 25 years and older (3.9%) and substantially lower than what has been previously reported during that same screening campaign in Kirehe district (8.4%), which hosts the Mahama camp.^9^ The prevalence of HCV among Mahama residents aged 15 years and older (1.1%) is similar to the prevalence among Rwandans aged 15–64 years who participated in the population-based survey (1.2%)^16^ but was lower than Rwanda's national prevalence among people aged 25 years and older who participated in the previous mass screening (6.8%).^10^ These statistics are not directly comparable due to the different ages of the screening populations—hepatitis C, in particular, is strongly associated with increased age in this setting—as well as because individuals who self-select into a mass screening campaign may suspect themselves to be at elevated risk of a disease compared to individuals selected at random to participate in nationally representative surveys. However, our findings clearly challenge earlier assumptions that the high prevalence of hepatitis in Kirehe district is attributable to refugee populations.^9^ While our research suggests that including refugee populations in national hepatitis prevention and management programs is a feasible way to promote health equity and further national hepatitis elimination plans, it is important to recognize that refugees may not be at higher risk for hepatitis than the general population in all settings. Our team strongly cautions against defaulting to a narrative in which refugee-focused screening campaigns are framed as a way to reach a population with elevated hepatitis prevalence relative to the host country because this framing can be stigmatizing and, as appears to be the case in Rwanda, may also be untrue. Instead, refugees should be included in national hepatitis programs in a way that promotes equitable access to health care and is neither stigmatizing nor discriminatory.

Our findings challenge earlier assumptions that the high prevalence of hepatitis in Kirehe district is attributable to refugee populations.

The cost per person screened was estimated at US$6.69, which is lower than the out-of-pocket cost for hepatitis screening among uninsured Rwandans (US$15.00) (Ministry of Health internal communication. This cost compares favorably to other public health interventions. For example, cervical cancer screening in sub-Saharan Africa is estimated to have a cost per woman screened ranging between US$3.33 and US$7.31 and cost per woman treated ranging between US$38 and US$71.^17^ Similarly, the cost per viral hepatitis case identified was US$136.20, which compares favorably to the annual cost of treatment for people with HIV in this setting (US$208).^18^ Although the costs reported in this article reflect only the cost of screening and do not include subsequent costs related to linkage to care and treatment, our team has previously found the per-client cost of HCV treatment in rural Rwanda, including linkage to care through a mobile clinic and medication, to be less than US$90.20. Although we did not conduct a formal cost-effectiveness analysis, the relatively low cost of this program suggests that it could be a feasible approach elsewhere.

We strongly recommend that future teams considering a similar campaign invest in strong stakeholder relationships and data collection tools. Coordination across national-, district- and field-level stakeholders was key for success. The involvement of camp leadership from the highest levels, including camp management, refugees' executive committee of the camp, up to the community level made the implementation successful. We also found that investing in high-quality electronic data capture resulted in better opportunities to assess the implementation of the program and track real-time progress. Since the close of the screening campaign, we have continued to use these data to contact clients who are diagnosed with chronic HBV and HCV for treatment assessment. Collecting high-quality data required close monitoring and daily, individualized feedback to data collectors early in the campaign.

### Limitations

The implementation of our campaign faced some limitations. First, approximately 9% of participants who screened positive for VL testing did not have their results returned, suggesting suboptimal communication with VL testing hubs. Second, while the school-based screening activities appear to have been an effective strategy to increase screening coverage among youth, our screening activities were suspended 2 weeks earlier than planned due to the coronavirus disease (COVID-19) pandemic, contributing to suboptimal coverage among youth. While coverage among women was 80% or higher for all women aged older than 20 years, additional outreach or weekend activities may be necessary to get adequate coverage among younger men, who often work outside of the camp. These programmatic challenges could lead to imprecision or bias in some of our estimates. For example, we would expect missing VL results to reflect random errors in administrative processes, such as misrecorded contact information or a lost sample, that would not be associated with the clients' VL status and would therefore lead to some imprecision but not systematic bias our estimates of VL positivity. However, because clients self-select into mass screening campaigns, it is possible that individuals who believed themselves to be at higher risk of hepatitis, for example, due to known family history, would be more likely to engage in screening than those who did not, which would lead to an overestimation of hepatitis prevalence. This pattern of higher hepatitis prevalence among individuals participating in mass screening campaigns than among the general population has been reported previously in Rwanda. However, in general, we would expect the impact of these processes on our estimates to be small due to the relatively low rates of missing VLs and high rates of screening coverage.

## CONCLUSION

Conducting a mass screening campaign for HBV and HCV was a feasible and effective way to integrate refugees into Rwanda's national health program. This campaign provided an opportunity to identify clients who were eligible for treatment assessment and to provide educational counseling on prevention measures among people who screened negative. This program, which relied on close collaboration with stakeholders and community members, can serve as a reference that could be duplicated in other refugee camps in Rwanda and other countries with similar settings.

## Translation

**En Français**

**Mise en œuvre de l'inclusion des réfugiés dans la campagne nationale de dépistage de l'hépatite virale B et de l'hépatite C dans le camp de réfugiés de Mahama, au Rwanda**

**Message clés**

Mener une campagne en masse pour le dépistage du VHB et du VHC était une stratégie faisable, efficace et peu coûteuse pour intégrer les réfugiés dans le programme national de prévention et de gestion de l'hépatite au Rwanda.

La coordination entre les parties prenantes au niveau national, du district et de la communautaire ainsi que l'analyse des données à temps sont essentielles pour mettre en œuvre une campagne bien coordonnée et de haute qualité enfin d'atteindre une couverture de dépistage élevé.

Cette campagne a été une occasion identifier les patients éligibles au traitement et de donner des conseils educatif sur les mesures de prévention pour des personnes dépistées négatives. Les décideurs politiques devraient explicitement inclure les réfugiés et autres personnes déplacées dans leurs stratégies nationales d'élimination et de contrôle de l'hépatite.

**Résumé**Introduction: L'Organisation Mondiale de la Santé (OMS) a lancé un appel pour l'élimination du virus de l'hépatite B (VHB) et du virus de l'hépatite C (VHC) d'ici 2030, en tant que menaces pour la santé publique. En réponse à la demande du Haut-Commissariat des Nations Unies pour les Réfugiés, le Rwanda a été le premier pays à inclure les réfugiés dans son programme national de prévention et de prise en charge des hépatites virales en 2019. Nous avons utilisé des données secondaires pour décrire la mise en œuvre du premier programme de dépistage du VHB et du VHC parmi les réfugiés au Rwanda.

Méthodes: Des tests de diagnostic rapide ont été utilisés pour dépister l'antigène de surface du VHB (HBsAg) et les anticorps du VHC (anti-VHC). Nous avons utilisé les données de routine recueillies lors de la campagne de dépistage en masse du VHB et du VHC parmi les réfugiés Burundais vivant dans le camp de Mahama et les données du programme pour estimer le taux du dépistage, la prévalence du VHB et du VHC et le coût de la campagne.

Résultats: Pendant 28 jours de février à Mars 2020, 26 498 individus ont été dépistées pour le VHB et le VHC, ce qui reflète une couverture de dépistage de 77,9% (intervalle de confiance [IC] à 95% = 76,5%, 78,4%). La couverture était supérieure à 90% chez les femmes âgées de 30 à 64 ans, mais les groupes de jeune âge et les hommes étaient moins susceptibles d'être dépistés. En moyenne, 946 clients ont été dépistés par jour. La prévalence des anti-VHC était de 1,1% (IC à 95% = 1,0%, 1,3%) et la prévalence de l'AgHBs était de 3,8% (IC à 95% = 3,6%, 4,0%). Nous estimons que le coût total de la campagne était de 177 336,60 USD, ce qui correspond à un coût par personne dépistée de 6,69 USD.

Conclusion: La mise en œuvre d'un dépistage en masse était une stratégie réalisable et efficace pour atteindre un taux de dépistage élevé et identifier les réfugiés éligibles au traitement du VHB et du VHC. Ce programme de dépistage dans le camp de réfugiés de Mahama peut servir de référence pour d'autres camps de réfugiés au Rwanda et ailleurs.

**Peer Reviewed**
